# Optimal translational fidelity is critical for *Salmonella* virulence and host interactions

**DOI:** 10.1093/nar/gkz229

**Published:** 2019-04-03

**Authors:** Yongqiang Fan, Laurel Thompson, Zhihui Lyu, Todd A Cameron, Nicholas R De Lay, Anne Marie Krachler, Jiqiang Ling

**Affiliations:** 1 College of Life and Health Sciences, Northeastern University, Shenyang 110819, People's Republic of China; 2 Department of Microbiology and Molecular Genetics, McGovern Medical School, University of Texas Health Science Center, Houston, TX 77030, USA; 3 Shenyang National Laboratory for Materials Science, Northeastern University, Shenyang 110819, People's Republic of China; 4 Department of Cell Biology and Molecular Genetics, The University of Maryland, College Park, MD 20742, USA

## Abstract

Translational fidelity is required for accurate flow of genetic information, but is frequently altered by genetic changes and environmental stresses. To date, little is known about how translational fidelity affects the virulence and host interactions of bacterial pathogens. Here we show that surprisingly, either decreasing or increasing translational fidelity impairs the interactions of the enteric pathogen *Salmonella* Typhimurium with host cells and its fitness in zebrafish. Host interactions are mediated by *Salmonella* pathogenicity island 1 (SPI-1). Our RNA sequencing and quantitative RT-PCR results demonstrate that SPI-1 genes are among the most down-regulated when translational fidelity is either increased or decreased. Further, this down-regulation of SPI-1 genes depends on the master regulator HilD, and altering translational fidelity destabilizes HilD protein via enhanced degradation by Lon protease. Our work thus reveals that optimal translational fidelity is pivotal for adaptation of *Salmonella* to the host environment, and provides important mechanistic insights into this process.

## INTRODUCTION

The accuracy of the proteome depends on faithful gene expression processes, including DNA replication, transcription, and translation (1–4). The average rate of gene expression errors accumulating from these steps is around 10^−4^ to 10^−3^, which is primarily contributed by errors from aminoacyl-tRNA synthesis (aminoacylation) and ribosomal decoding during translation (3,5,6). To limit the errors and maintain translational fidelity, the protein synthesis machinery utilizes multiple quality control mechanisms. In addition to the initial substrate selection, proofreading is used during both aminoacylation (7–10) and ribosomal decoding (11,12). Despite such extensive quality control mechanisms, translational fidelity is still frequently perturbed by genetic variations and environmental cues. A survey of natural *Escherichia coli* isolates revealed that ribosomal fidelity varies over 10-fold among different genetic backgrounds (13). Mutations in ribosomal proteins (14–16), 16S ribosomal RNA (17), transfer RNAs (18), aminoacyl-tRNA synthetases (6,7) and tRNA modification enzymes (19) have all been shown to either increase or decrease overall translational fidelity. Apart from these permanent genetic changes, environmental stresses such as antibiotics (20), oxidative stress (21,22), and nutrient starvation (23) also lead to a transient increase in translational errors.

Given the flexibility of translational fidelity, an intriguing question that arises is how translational errors affect cells. For decades, translational errors were thought to be harmful and it was assumed these errors needed to be avoided to increase fitness. For example, severe translational errors induced by aminoglycoside antibiotics cause bacterial cell death (24,25), and a mutation in alanyl-tRNA synthetase leads to amino acid misincorporation and neurodegeneration in mice (26). Nevertheless, more recent studies show that certain types of translational errors may indeed improve fitness by protecting cells from oxidative damage and antibiotics (27–30). It has now become increasingly clear that the effects of translational errors on fitness may depend both on the cellular context and the surrounding environment (30), yet we are only beginning to understand the underlying mechanisms. In particular, the role of translational fidelity in bacterial pathogenesis is largely unexplored. Results from a previous study suggest that increasing translational fidelity affects the virulence of *Salmonella* in mice (15). However, this work used the LT2 strain background, which is avirulent due to a mutation in *rpoS* and not suitable to study virulence or bacteria-host interactions (31,32). In the present work, we have used the fully-virulent *Salmonella enterica* Typhimurium strain ATCC 14028 (referred to as wild-type or WT) to investigate how altering translational fidelity affects bacterial virulence and the underlying molecular mechanisms driving host interactions.

*Salmonella* are enteric pathogens that cause enteric fever and gastroenteritis, leading to 100 million infections and more than 300,000 deaths in humans each year (33). *Salmonella* contains multiple pathogenicity islands (SPIs) that encode critical virulence genes (34). Two conserved SPIs (SPI-1 and SPI-2) encode separate Type III Secretion Systems (T3SS) and multiple effector proteins (35,36). SPI-1 genes are critical for invasion of host cells, whereas SPI-2 genes are important for survival and replication within host cells (37). Our results here show that perturbing translational fidelity decreases expression of *Salmonella* virulence factors, in particular SPI-1 genes, and leads to defects in host cell invasion. This suggests that an optimal translational fidelity is critical for *Salmonella* to interact with hosts.

## MATERIALS AND METHODS

### Bacterial strains and growth conditions

All strains used in this study are listed in Supplementary Table S1. The *Salmonella* strains were derivatives of *Salmonella enterica subsp. enterica serovar* Typhimurium (ATCC^®^ 14028™), which was obtained from American Type Culture Collection. Unless otherwise noted, all bacteria used in this study were cultured in regular Lennox LB Broth (tryptone 10 g/l, sodium chloride 5 g/l, yeast extract 5 g/l) at 37°C. For typical experiments, overnight cultures of *Salmonella* strains were diluted into fresh LB medium with inoculation OD_600_ of 0.05, and were grown aerobically at 37°C to mid-log phase (OD_600_ ∼ 0.6–0.8) or early-stationary (OD_600_ ∼ 1.2–1.5).

### Plasmids and genome engineering of bacterial strains

All plasmids used in this study are listed in Supplementary Table S1. The plasmids in this study were constructed with the In-Fusion HD Cloning Kit (Clontech) according to manufacturer's instruction. The promoter regions of *hilA* and *hilD* contain sequences of 1090 bp and 315 bp upstream of the start codons. AG1 vector was used to make genes overexpression. pZS-*P_tet_-yfp*-Spc was constructed by replacing the ampicillin resistance marker in pZS*11 with the spectinomycin (Spc) resistance cassette from the plasmid pCDF. pZS-*P_tet_-mCherry*-Chl was constructed by replacing the *yfp* gene in pZS*11 with *mCherry*, and replacing the ampicillin resistance gene with a chloramphenicol (Chl) resistance cassette. pZS-*thyA* was created by replacing the *yfp* region in pZS*11 with the open reading frame of *thyA* amplified from the *Salmonella* genome.

The rpsD* and rpsL* mutants were generated with a modified multiplex automated genome engineering (MAGE) method (38). Arabinose-induced lambda-Red recombinase expressed from plasmid pKD46 was used in this study. Oligos 5′-T*G*T*G*TCC TCTCTTTGGTACTAAGCTTTACTTGGAGTAAAGCTCGACGTTAAGGTGTTCGTTAATGTCCGCAGACAGATCAGAACGCTCAG-3′ and 5′- T*C*A*G*ACGAACACGGCAAACTTTACGCAGTG CGGAGTTCGGTTTGTTTGGAGTGGTAGTATATACACGAGTACATACGCCACGTTTTTGCG-3′) was used to introduced *rpsD* I199N (rpsD*) and *rpsL* K42N (rpsL*) mutations, respectively. The plasmid was cured by incubation of cells at a non-permissive temperature (37°C) after the mutations were introduced. The mutations were confirmed by both polymerase chain reaction (PCR) and Sanger sequencing.

HilD was C-terminally epitope tagged with a 3× FLAG tag, according to the method described in (39) with some modifications. Briefly, the native *thyA* gene was deleted from the *Salmonella* strain containing pKD46 plasmid with a Chl resistance cassette. A *thyA* cassette with a *tet* promoter and *rrnB* T1 terminator was amplified from plasmid pZS-thyA. The *thyA* cassette was integrated into the C-terminus of HilD on the chromosome, and replaced with the *hilD-FLAG* gBlock (Integrated DNA Technology, Illinois, USA). Finally, the native *thyA* gene was recovered. The constructs were verified by sequencing. The C-terminal FLAG tagged HilD has been previously shown to be fully functional (40).

All in-frame gene deletion mutants were constructed as described (41) using Chl as the resistance marker. All the mutants were verified by PCR, and the antibiotic resistance genes were subsequently removed from the deletion strains using the plasmid pCP20. The marker-free deletion strains were verified by both loss of resistance and PCR.

The *P_tet_*-mCherry-Chl and *P_tet_*-yfp-Spc cassettes were amplified from the plasmids pZS*-*P_tet_*-mCherry-Chl and pZS*-*P_tet_*-yfp-Spc by PCR, respectively. The cassettes were integrated into the chromosomal region of the WT, rpsD*, and rpsL* strains between genomic coordinates 3115468 and 3115743 by induction of Red recombinase, and the positive clones were selected by chloramphenicol or spectinomycin resistance, respectively.

### Determination of mistranslation and protein synthesis rates

The pZS-*P_tet_*-m-TGA-y plasmid was used to determine the mistranslation rates as described (42), with plasmid pZS-*P_tet_*-m-y as the control. To determine the protein synthesis rate, the mCherry data from pZS-P*_tet_*-m-y plasmid was used. The protein synthesis rates were calculated as described (43).

### RNA sequencing and data analysis

Overnight cultures of WT, rpsD* and rpsL* strains were diluted into 5 ml of fresh LB medium in 50 ml flasks with inoculation OD_600_ of 0.05, and were grown at 37°C to mid-log phase (OD_600_ ∼ 0.6–0.8) or early-stationary (OD_600_ ∼ 1.2–1.5) phase for RNA extraction. Total RNA was prepared using the RNAprotect Bacteria Reagent and RNeasy Protect Bacteria Kit (Qiagen, Valencia, CA, USA) according to the user's manuals. Library preparation and Illumina sequencing were performed by GENEWIZ (South Plainfield, NJ, USA). Three biological replicates of each strain per condition were sequenced.

High-throughput RNA sequencing data were preprocessed for alignment with Cutadapt (44) to remove Illumina sequencing adapters and low-quality bases (PHRED < 20) from the ends of reads. Trimmed read pairs were then aligned to the NCBI GenBank assembly GCA_000022165.1 for *S. enterica* serovar Typhimurium 14028 using Bowtie2 (45), and the resulting SAM files were converted to BAM files using SAMtools (46). Aligned reads were quantified to individual genes in the annotated genome using Cufflinks (47) and differential expression analysis among samples was performed with Cuffdiff (47) using multi-read correction and FPKM library normalization. These analyses were performed using high-performance computing resources of the Texas Advanced Computing Center (TACC) at The University of Texas at Austin. Pathway enrichment analyses was conducted using Gene Ontology (http://geneontology.org/page/go-enrichment-analysis).

### Quantitative reverse transcription-PCR

For detection of gene expression from the SPI-1 locus, mid-log phase cells (OD_600_ ∼ 0.6–0.8) grown in LB medium at 37°C were collected. Total RNA was extracted using the hot phenol method and residual chromosomal DNA was removed as previously described (48). Reverse transcription and PCR were performed using the iScript cDNA Synthesis Kit and the SsoAdvanced Universal SYBR Green Supermix kit (Bio-Rad, Hercules, CA, USA) according to the manufacturer's instructions. The *mreB* gene, which encodes a homolog of the eukaryotic actin protein, was used as an internal reference for normalization. The ΔΔCt method was used to calculate the fold changes of target genes in the mutants compared with the WT strain.

### Swimming motility assay

Overnight cultures of bacteria were diluted 1:100 into fresh LB and grown to mid-log phase at 37°C with gentle agitation. All cultures were normalized to the same OD_600_ before being spotted onto the fresh soft agar plates (10 g/l of tryptone, 5 g/l of NaCl and 2.5 g/l agar). The plates were incubated at 37°C for 4 h and the diameters of the bacterial spots were measured. The quantitative results represent the percentage of the diameter compared to that of the WT strain on the same plate.

### Attachment to and invasion of macrophage and epithelial cells

The J774A.1 macrophage cell line (ATCC^®^ TIB-67™) and the RKO epithelial cell line were cultured separately in cell culture dishes in Dulbecco's minimum essential medium (DMEM, Sigma) supplemented with 10% fetal bovine serum (FBS, sigma) and 1% penicillin/streptomycin at 37°C with 5% CO_2_. Cells were collected when they reached 80% confluence, and 200 μl DMEM cell culture medium containing 5 × 10^4^ cells was seeded in each well of 96-well cell culture plates. The cell culture plates were incubated for 18 h at 37°C in a CO_2_ incubator before use. The bacteria cells were grown in LB medium into early-stationary phase before mixing with host cells. The infection was conducted with multiplicity of infection (MOI) of 100, with the bacteria resuspended in 200 μl DMEM medium. The plates were centrifuged at 500 × g for 5 min, and incubated at 37°C in a CO_2_ incubator for 40 min. The free bacteria cells were then removed, and mammalian cells were washed three times with PBS. For the attachment assay, 100 μl PBS with 0.1% triton was used to lyse the host cells, and the numbers of viable bacterial cells were determined by colony forming units (CFU). For invasion, the samples were treated with 200 μl DMEM containing 100 μg/ml gentamycin for 1 h at 37°C in a CO_2_ incubator. After washing with PBS 3 times, host cells were lysed with 100 μl PBS containing 0.1% triton, and the numbers of viable bacterial cells were determined by CFU.

### β-Galactosidase assays

Cells were grown in 1 ml LB supplemented with different concentrations of isopropyl β-d-1-thiogalactopyranoside (IPTG) at 37°C to an OD_600_ of 0.7–1.0. Four biological repeats were conducted for each sample. 100 μl culture was transferred to each well of 96-well plate, and the OD_600_ values were determined with a microplate reader. 2 μl of 0.1% SDS and 2 μl of chloroform were added into each well and incubated on the bench for 20 min. Measurement of β-galactosidase was initiated by addition of 50 μl of 4 mg/ml *o*-nitrophenyl-β-d-galactopyranoside (ONPG) in 0.1 M phosphate buffer (pH 7.0) that contains 2 mM MgSO_4_ and 10 mM 2-mercaptoethanol. The plate was then put into a microplate reader to record the OD_420_ and OD_550_ values over 1 h every 3 min. Assay units were calculated as 1000× slope of (OD_420_ – 1.75 × OD_550_)/OD_600_.

### Determination of protein expression and degradation

To determine the protein level of HilD and Lon, cells from early-stationary phase were collected and washed once with phosphate buffer before sonication to lyse the cells. To determine the degradation rate, 100 μg/ml chloramphenicol was added to the culture to stop translation at time zero. Western blot was performed according to standard procedures using a primary anti-FLAG antibody or an antibody against Lon.

### Protein secretion assay

*Salmonella* strains were cultured in 15 ml LB to early-stationary phase (OD_600_ ∼ 1.2). The supernatant was collected by filtering through 0.45 μm PVDF membranes, and concentrated with Amicon Ultra-4 centrifugal filters (10 K). The samples were then separated by SDS-PAGE and proteins visualized with Pierce silver stain kit according to manufacturer's protocol.

### Zebrafish maintenance and breeding

The zebrafish (*Danio rerio)* lines used in this study were AB wild-type fish and transgenic fish of the Tg(*mpo::egfp*)^i114^ line that produce green fluorescent protein (GFP) in neutrophils (49). Adult fish were kept in a recirculating tank system at the UTHealth Center for Laboratory Animal Medicine and Care under conditions of a 14 h/10 h light/dark cycle at pH 7.5 and 26°C. Zebrafish care and breeding and experiments were performed in accordance with the Guide for the Care and Use of Laboratory Animals, and have been approved by the Institutional Animal Welfare Committee of the University of Texas Health Science Center, protocol number AWC-16-0127. Eggs were obtained from natural spawning between adult fish, which were set up in groups of seven (four females and three males) in separate breeding tanks. After collection of eggs, larvae were kept in a diurnal incubator under conditions of a 14 h/10 h light-dark cycle with the temperature maintained at 28–29°C. Embryos were raised in petri dishes containing E3 medium (5 mM NaCl, 0.17 mM KCl, 0.33 mM CaCl_2_, 0.33 mM MgSO_4_) supplemented with 0.3 μg/ml of methylene blue. From 24 hpf, 0.003% 1-phenyl-2-thiourea (PTU) was added to prevent melanin synthesis. During infections, larvae were maintained at 32°C. All zebrafish care and husbandry procedures were performed as described previously (50).

### Maintenance of *Paramecium caudatum*

Paramecia were cultured at 22°C in a 25 ml cell culture flask with E3 medium containing *E. coli* K-12 MG1655 as food source. To maintain the culture, 1 ml of an existing paramecium culture was passaged into 9 ml of fresh E3 medium containing 10^9^ CFU/ml of *E. coli* cells.

### Clearance of *Salmonella enterica serovar Typhimurium* by *P. caudatum*

To determine *Salmonella* viability within *P. caudatum*, samples were removed from *P. caudatum* cultures at indicated time points, lysed with 1% Triton X-100 in PBS, homogenized and plated on LB plates for CFU counting.

### Foodborne and waterborne fish infections

For infection experiments, bacterial cells at early-stationary phase were harvested by centrifuging at 8000 rpm for 5 min and adjusted to an OD_600_ of 1.0 (approximate concentration of 1 × 10^9^ bacteria/ml). *P caudatum* numbers were quantified using a hemocytometer and normalized to a concentration of 7 × 10^4^ paramecia/ml, and the reaction mixtures in a six-well plate were incubated for 2 h at 32°C. Following pre-incubation, paramecia were washed four times with E3 and co-incubated with zebrafish larvae (5 dpf) in six-well plates for 2 h or indicated time and continued for foodborne infection. For waterborne infections, the overnight *Salmonella* cultures were normalized to an OD_600_ of 1.0 in E3 medium, and added directly to zebrafish larvae (5 dpf). Following infections, zebrafish larvae were washed with E3, either anesthetized by adding tricaine to a concentration of 160 μg/ml for imaging or euthanized with 1.6 mg/ml tricaine for determination of bacterial burden.

### Imaging of infected fish

Anesthetized zebrafish larvae were positioned in a 96-well glass-bottom black plates and immobilized with 1% low-melting-point agarose solution containing 160 μg/ml tricaine. 200 μl E3 medium containing 160 μg/ml tricaine was used to cover the immobilized larva. Live imaging was performed at 32°C and 80% humidity. An Olympus inverse confocal microscope equipped with a 10× objective was used for acquisition of two fluorescent channels and one differential interference contrast (DIC) channel. The three-dimensional (3D) images and the 4D images produced by the time-lapse acquisitions were processed, clipped, examined, and interpreted using ACQUISIITON FV31S-SW software (Olympus). Maximum intensity projection was used to project developed *Z*-stacks, and files subjected to deconvolution were exported in tiff format for images.

### Determination of bacterial burden in infected fish

After euthanasia, larvae were transferred to individual micro centrifuge tubes and washed once with E3. 120 μl pronase (Roche, 1 mg/ml) in PBS was used to resuspend the fish, and samples were vortexed thoroughly following a 1 h incubation at 22°C. The larvae were then homogenized by passing through fine needles (31 gauge). 50 μl lysate was serially diluted and used to determine the bacterial burden. Bacteria on the selective plates were incubated overnight at 37°C before CFU counting.

## RESULTS

### Perturbing translational fidelity down-regulates expression of virulence genes

We chose *Salmonella enterica* Typhimurium as a model pathogen to understand the role of translational fidelity in bacterial pathogenesis. To alter the translational fidelity of *S*. Typhimurium, we used a genome editing tool (38) to introduce point mutations (I199N and K42N) in the ribosomal genes *rpsD* and *rpsL* in the virulent strain ATCC 14028. Previous studies have shown that the *rpsD* I199N mutation increases various translational errors in *Salmonella* (15) and *E. coli* (16), and the *rpsL* K42N mutation decreases translational errors, such as stop-codon readthrough and missense errors (15,51). The *Salmonella* and *E. coli* ribosomes are highly similar with almost 100% sequence identity (99.5% for RpsD and 100% for RpsL). We used a convenient and accurate dual-fluorescence reporter readthrough assay (42) to validate the error rate of our mutant strains. Compared with the WT, our resulting *Salmonella* strains *rpsD* I199N (rpsD*) and *rpsL* K42N (rpsL*) strains displayed increased and decreased rates of UGA readthrough, respectively (Supplementary Figure S1A), which is consistent with previous work (15,16,51). The mutations exhibited little effect on growth (Supplementary Figure S1B and C) and colony morphology (S1D). In addition, the protein synthesis rates determined using a yellow fluorescent reporter did not decrease in the mutant strains (Supplementary Figure S1B).

**Figure 1.**
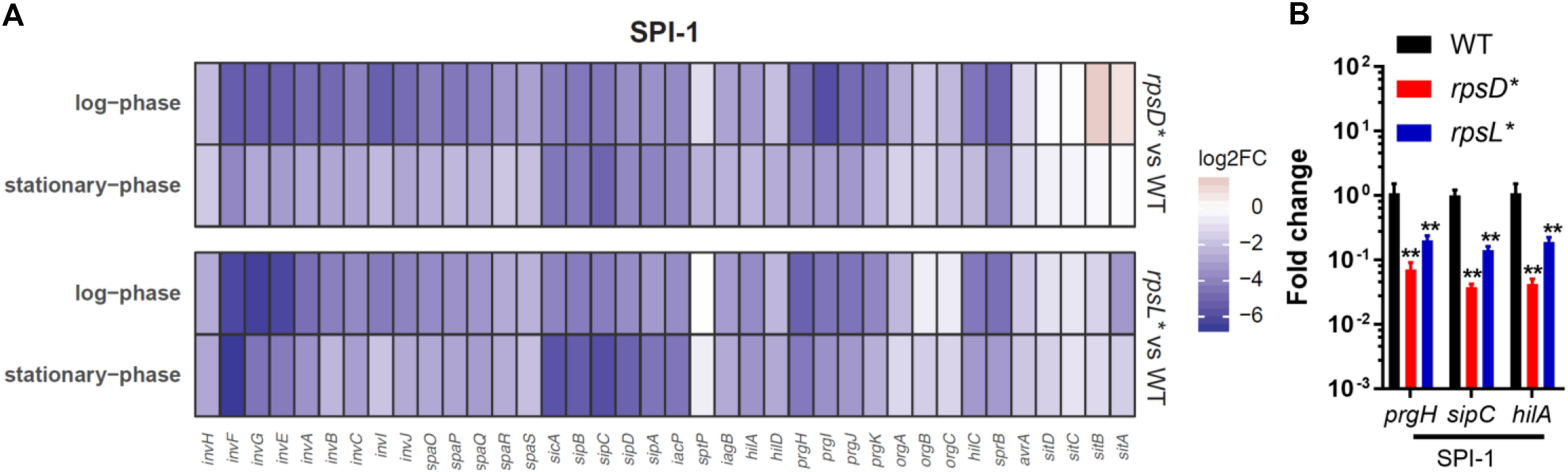
Down-regulation of virulence genes in rpsD* and rpsL* strains. WT, rpsD* and rpsL* Salmonella cells were grown to mid-log (OD_600_ ∼ 0.8) or early-stationary phase (OD_600_ ∼ 1.2) in LB media at 37°C. The total mRNA was extracted and subjected to RNA sequencing (**A**) and qRT-PCR (mid-log phase, **B**). (A) shows the mRNA profile of known SPI-1 genes. The majority of SPI-1 genes were substantially down-regulated in both the rpsD* and rpsL* strains. Three biological replicates were used for RNA-Seq, and *n* = 6 for qRT-PCR. **P < 0.01 determined using one-way analysis of variance (ANOVA). Error bars represent one standard deviation.

To assess the global effects of altering translational fidelity in *Salmonella*, we next performed RNA sequencing (RNA-Seq) analysis of the WT, rpsD*, and rpsL* cells grown in Lennox broth (LB) to mid-log or early-stationary phase (Supplementary Tables S2 and S3). Gene Ontology enrichment analysis revealed that pathogenesis was the most significantly down-regulated pathway in both the rpsD* (*P* value 2.5 × 10^−27^) and rpsL* (*P* value 1.9 × 10^−15^) strains compared with the WT at early-stationary phase (Supplementary Table S4), and was also among the most significantly down-regulated pathways in rpsD* (*P* value 1.6 × 10^−26^) and rpsL* (*P* value 7.3 × 10^−19^) at mid-log phase (Supplementary Table S5). Other major down-regulated pathways include flagellar motility and protein secretion (Supplementary Tables S4 and S5). Almost all SPI-1 genes were significantly down-regulated in both the error-prone rpsD* and high-fidelity rpsL* strains (Figure 1A, Supplementary Tables S2 and S3). We further selected three SPI-1 genes (*prgH, sipA*, and *hilA*) shown by RNA-Seq to be down-regulated in the rpsD* and rpsL* strains, and confirmed their down-regulation with real-time quantitative reverse-transcription PCR (qRT-PCR) (Figure 1B). These results suggest that optimal translational fidelity is critical for expression of *Salmonella* virulence genes.

### Non-optimal translational fidelity impairs host-cell interactions and animal infection

**Figure 2.**
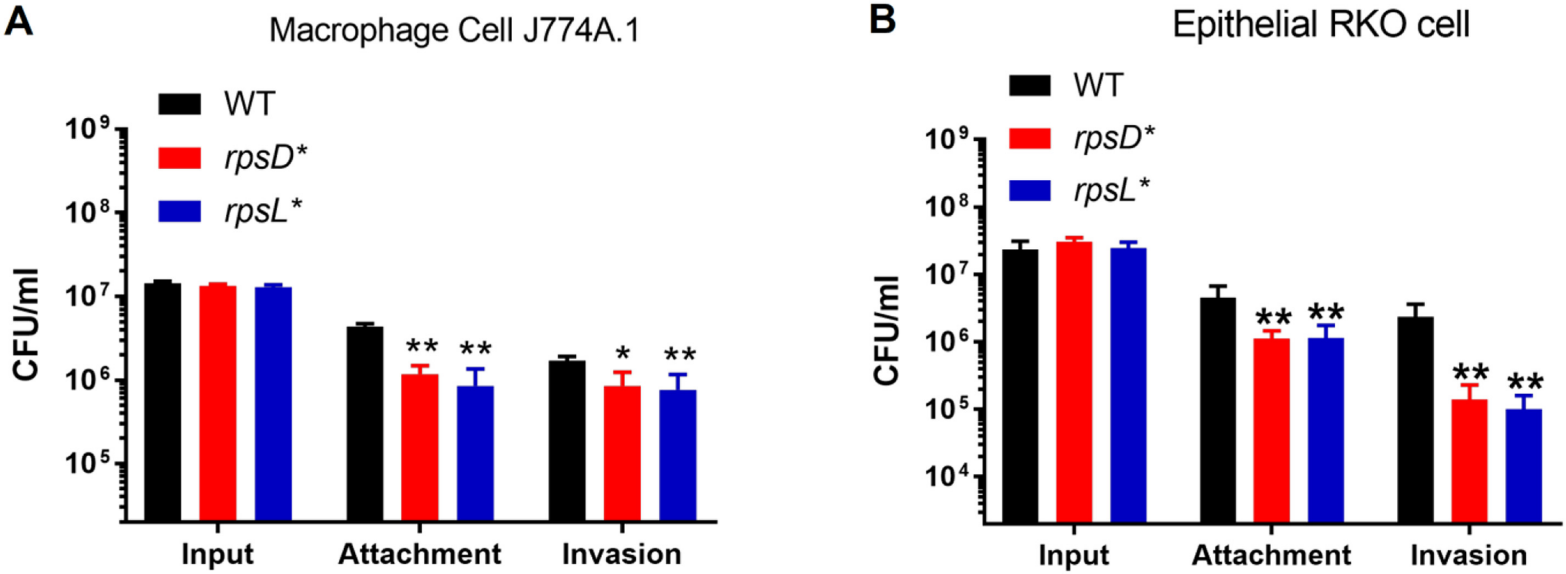
Non-optimal translational fidelity in *Salmonella* impairs host-cell attachment and invasion. *Salmonella* cells were grown to early-stationary phase in LB media at 37°C and incubated with cultured macrophage (**A**) and epithelial (**B**) cells. *n* = 8. *P < 0.05; **P < 0.01 determined using one-way ANOVA. Error bars represent one standard deviation.

*Salmonella enterica* Typhimurium is an enteric pathogen that infects a broad range of animal hosts (35,52). The SPI-1 T3SS transports effector proteins that are critical for *Salmonella* to invade host cells and cause inflammation (35,52). Our RNA-Seq results show that many critical SPI-1 effector genes, such as *sipC* and *sopB*, are down-regulated over 10-fold in both the error-prone rpsD* and high-fidelity rpsL* strains (Figure 1 and Supplementary Table S2), prompting us to test the interactions of *S*. Typhimurium strains with host cells. We observed that both rpsD* and rpsL* strains were defective in attachment and invasion of macrophage and epithelial cells (Figure 2), in line with down-regulation of SPI-1 T3SS and related effector genes.

**Figure 3.**
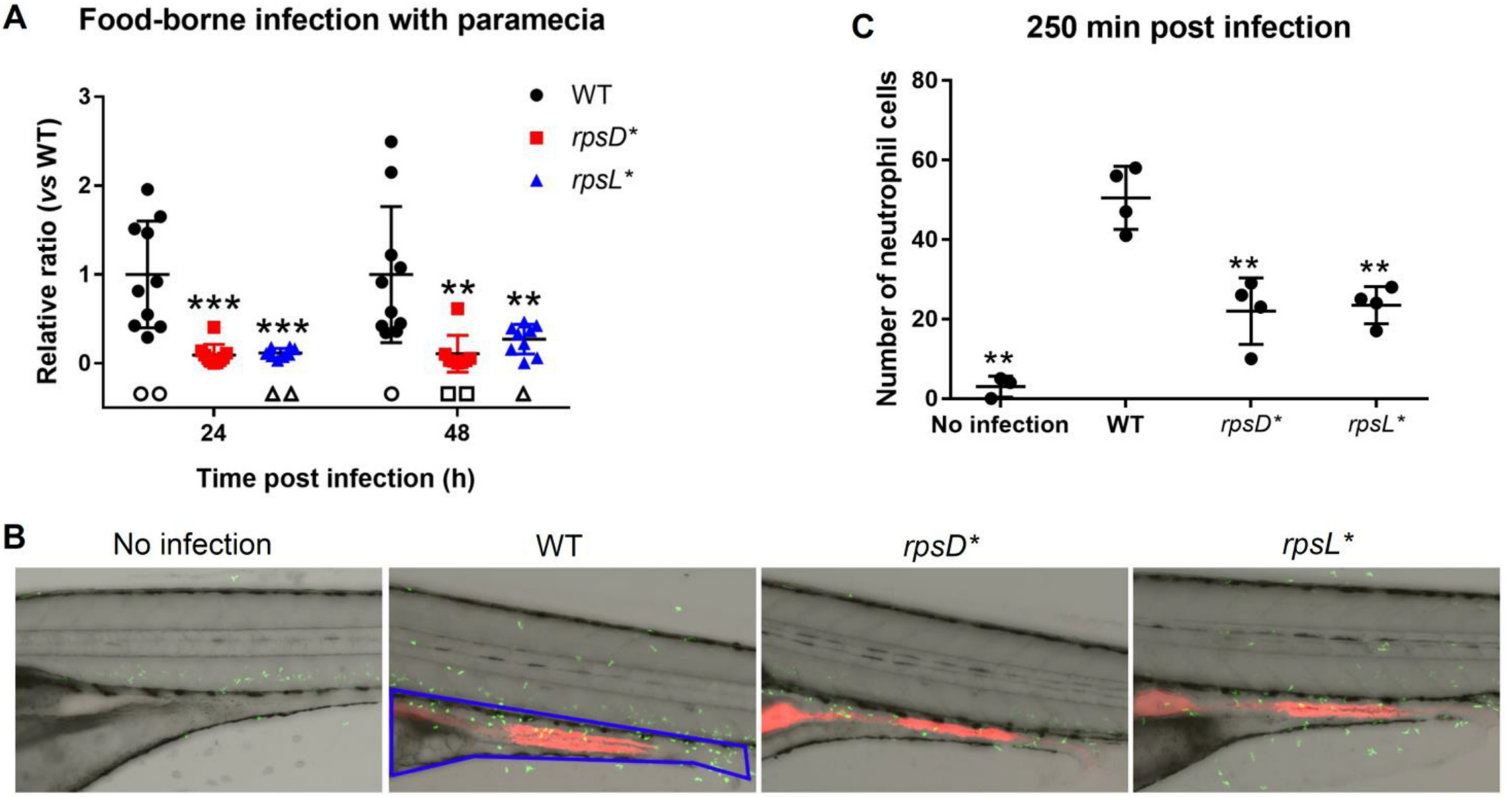
Non-optimal translational fidelity in *Salmonella* impairs zebrafish infection. (**A**) Pairs of WT/WT, WT/rpsD* and WT/rpsL* cells (grown to early-stationary phase in LB at 37°C) were mixed at equal numbers and delivered to zebrafish via paramecia. The colony forming units were determined 24 and 48 h post-infection. Empty symbols indicate that no *Salmonella* cells were recovered from the fish and thus no ratio was calculated. 10 fish were used for each pair of *Salmonella* strains at each time point. (**B**) WT, rpsD* and rpsL* cells carrying mCherry (red fluorescence) were delivered to the gut of zebrafish via food-borne infection. Zebrafish are Tg(*mpo::egfp*)^i114^ (neutrophils are green fluorescent). The blue line indicates the gut area. 4 fish were used for each *Salmonella* strain. (**C**) Quantitation of the number of neutrophils recruited to the gut by WT, rpsD* and rpsL* cells. Each dot in A and C represents one fish. The average *Salmonella* CFU recovered from each fish at 24 and 48 h post infection is 4 × 10^3^ and 3 × 10^4^, respectively. **P < 0.01; ***P < 0.001 determined using one-way ANOVA. Error bars represent one standard deviation.

Next, we tested how altered translational fidelity would impact *in vivo* fitness using a larval zebrafish vertebrate model. The zebrafish has become an important model to study bacterial pathogenesis *in vivo*, and can provide mechanistic insights into the processes that drive host-microbe interactions (53,54). In contrast to mouse models, *S*. Typhimurium infection of zebrafish is limited to the gastrointestinal tract, and can be studied against the backdrop of the endogenous microbiota (55). We adapted a recently developed food-borne intestinal colonization model to deliver an equal number of WT and challenge *Salmonella* cells (WT, rpsD*, or rpsL*) via the protozoan *Paramecium caudatum* (56). The prey vehicle is taken up and rapidly digested by the larval fish, releasing the bacteria into the gut where they proceed to colonize the intestinal mucosae (Figure 3).

After 24 and 48 h, zebrafish were homogenized and the lysates were plated on selective media to determine the colony forming units (CFU) of WT and competing strains. Both rpsD* and rpsL* strains were outcompeted by the WT at 24 and 48 h (Figure 3A). This was not due to differences in bacterial stability within the *Paramecium* vehicle (Supplementary Figure S2A), and similar results were obtained following infection via bath immersion rather than food-borne infection (Supplementary Figure S2B).

*Salmonella* infection induces a strong pro-inflammatory response in the host, which the pathogen exploits to outcompete the endogenous microbiota to promote intestinal colonization (57,58). As such, recruitment of phagocytic cells to the site of infection is a hallmark of pathogenesis (59,60). To test the host inflammatory response following *Salmonella* infection, we measured recruitment of neutrophils into the zebrafish intestine over time using the transgenic line Tg(*mpo::egfp*)^i114^ that produces green fluorescent protein in neutrophils (49). Upon infection with WT *S*. Typhimurium, neutrophils were attracted to the fish intestine, reaching a peak around 250 min post infection (Figure 3B, C and Supplementary Figure S3). However, the rpsD* and rpsL* strains recruited significantly fewer neutrophil cells than the WT, suggesting that they elicit a less robust pro-inflammatory response.

### Non-optimal translational fidelity attenuates SPI-1 expression via HilD

**Figure 4.**
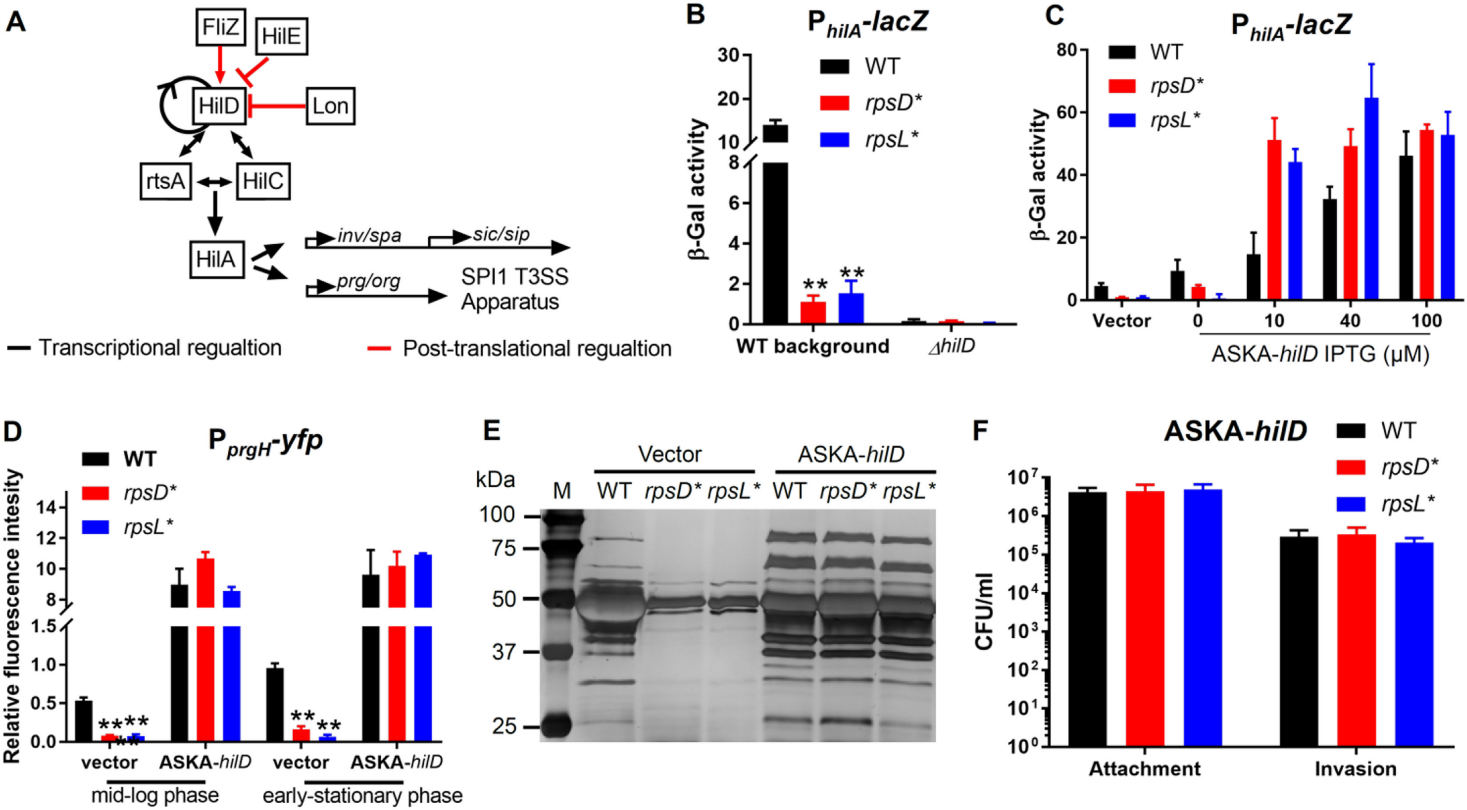
Down-regulation of SPI-1 genes by rpsD* and rpsL* mutations depends on HilD. (**A**) Expression of SPI-1 genes are regulated by the transcriptional activator HilA, which is transcriptionally activated by HilD, RtsA, and HilC. HilD positively regulates its own transcription. At the post-translational level, HilD is activated by FliZ, and negatively regulated by Lon and HilE. (**B**) A *lacZ* reporter is under the control of the *hilA* promoter and expressed in *Salmonella* strains. The *hilA* promoter activity is reduced in the rpsD* and rpsL* strains. Deleting *hilD* abolishes *hilA* expression in all tested strains. *Salmonella* cells were grown to mid-log phase in LB at 37°C. *n* = 4. (**C**) Overexpression of HilD fully restores *hilA* expression in the rpsD* and rpsL* strains. IPTG induces expression of HilD under the control of a *lac* promoter. *n* = 4. (**D**) A *yfp* reporter is under the control of the *prgH* promoter and the YFP signal is normalized with that of mCherry under the control of a constitutive *tet* promoter on the same plasmid (pZS*11). The *prgH* promoter activity is reduced in rpsD* and rpsL* cells at mid-log and early-stationary phases, and fully restored by overexpression of HilD (induced by 10 μM IPTG). (**E**) *Salmonella* cells were grown to early-stationary phase in 15 ml LB at 37°C. The supernatant was concentrated 100-fold and loaded on SDS-PAGE gels. Silver staining was performed to visualize the secreted proteins. This figure is a representative of three biological repeats. (**F**) *Salmonella* cells overexpressing HilD (induced by 10 μM IPTG) were grown to early-stationary phase in LB media at 37°C and incubated with cultured macrophage. *n* = 4. **P < 0.01 determined using one-way ANOVA. Error bars represent one standard deviation.

The master regulator of SPI-1 genes that control host cell attachment and invasion is the transcriptional activator HilA (61) (Figure 4A). Our RNA-Seq results revealed that the mRNA levels of *hilA* were reduced 5–13-fold in both rpsD* and rpsL* cells at mid-log and early-stationary phases (Supplementary Tables S2 and S3). Using a *lacZ* reporter under the control of the *hilA* promoter (P*_hilA_-lacZ*), we found that the promoter activity of *hilA* was decreased ∼10-fold in the rpsD* and rpsL* cells compared with the WT (Figure 4B). Transcription of a downstream SPI-1 gene *prgH* was also down-regulated 5-fold in both strains in regular and high-salt LB media (Supplementary Figure S4). HilA is positively regulated by HilD, HilC, and RtsA at the transcriptional level (62). Deleting *hilD* completely abolished the promoter activity of *hilA*, suggesting that HilD is an essential positive regulator of *hilA* transcription in *Salmonella* Typhimurium. HilD has been previously shown to directly regulates its own transcription (62). Our RNA-Seq result indicated that the mRNA level of *hilD* was decreased 4–6 fold in rpsD* and rpsL* (Supplementary Tables S2 and S3). In line with this, we used a transcriptional reporter to show that deleting *hilD* decreased its own promoter activity (Supplementary Figure S5). Therefore, positive transcriptional autoregulation is at least partially responsible for the decreased levels of *hilD* mRNA (Supplementary Tables S2 and S3) and transcription (Supplementary Figure S5) in rpsD* and rpsL*. To further test whether a HilD deficiency causes down-regulation of SPI-1 genes in the rpsD* and rpsL* strains, we overexpressed HilD on a plasmid under the control of a *lac* promoter. Overexpression of HilD fully restored *hilA* and *prgH* promoter activities in rpsD* and rpsL* (Figure 4C and D). Furthermore, overexpressing HilD rescued the defects of rpsD* and rpsL* in protein secretion and macrophage invasion (Figure 4E and F).

**Figure 5.**
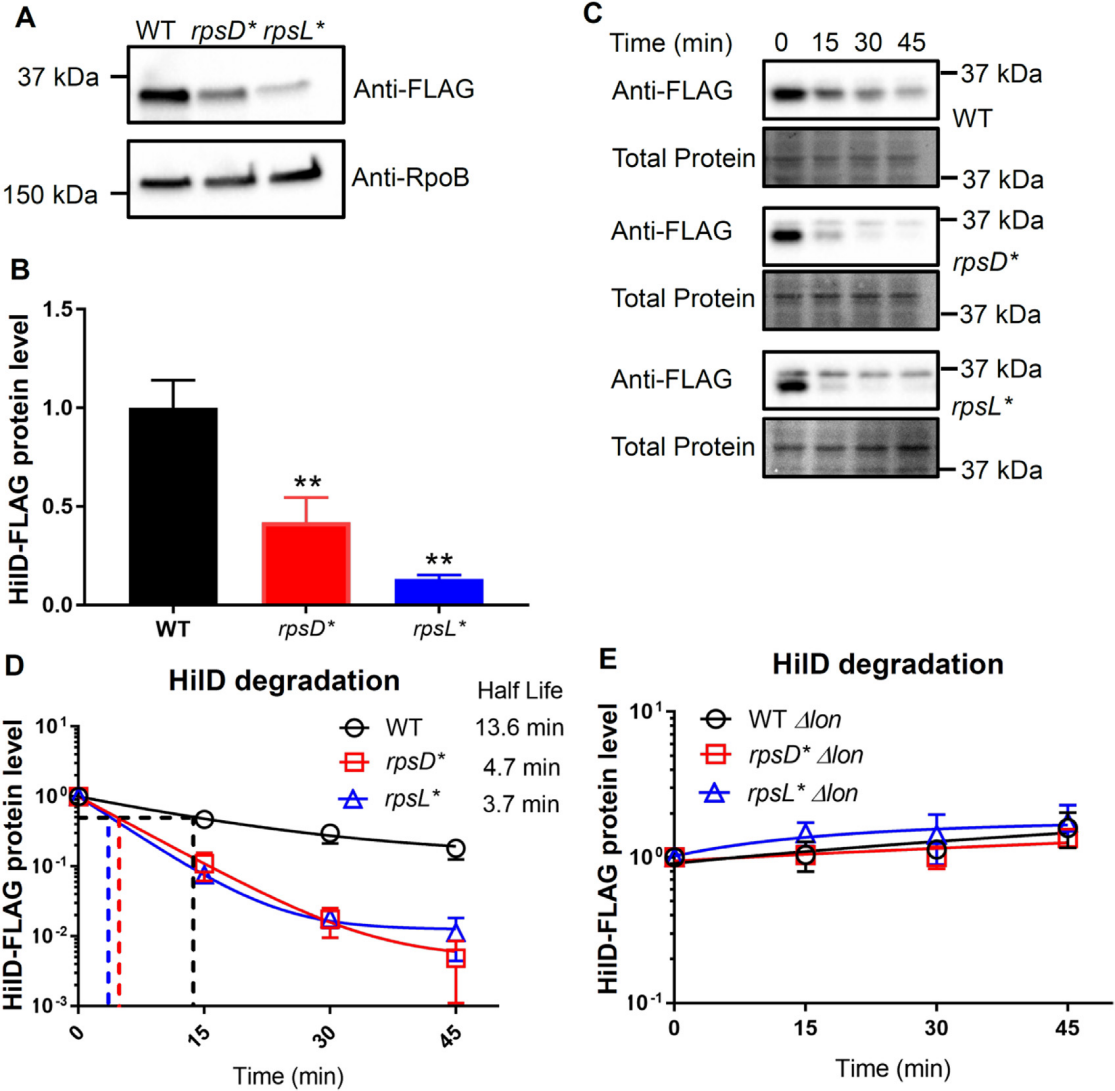
Increased and decreased translational fidelity both enhance degradation of HilD. (**A, B**) A FLAG tag was inserted at the 3′end of the native *hilD* gene and the HilD protein levels were probed with an anti-FLAG antibody using Western blotting from early-stationary phase cells grown in LB at 37°C. Both rpsD* and rpsL* mutations significantly reduced the HilD protein level at early-stationary phase. The HilD protein level was normalized with the control protein RpoB. (**C, D**) Protein synthesis was inhibited at 0 min with chloramphenicol, and degradation of HilD was monitored over the time course. HilD was degraded 3 times faster in both the rpsD* and rpsL* strains compared with the WT. (**E**) Western blotting against HilD-FLAG over time. Deleting *lon* abolished degradation of HilD in the WT, rpsD*, and rpsL* strains, suggesting that the Lon protease is primarily responsible for degradation of HilD in *S*. Typhimurium. *n* = 3. **P < 0.01 determined using one-way ANOVA. Error bars represent one standard deviation.

Next, we inserted a FLAG tag at the C-terminus of HilD in the native chromosomal locus and determined its protein level of HilD. The *hilA* transcription reporter assay demonstrated that the FLAG-tagged HilD was functional *in vivo* (Supplementary Figure S6). Western blotting analysis revealed that the protein level of HilD was significantly decreased in the rpsD* and rpsL* strains compared with the WT (Figure 5A and B). We further observed that compared with the WT, degradation of HilD was approximately 3 times faster in the rpsD* and rpsL* strains (Figure 5C and D), contributing to the lower level of HilD protein in these strains. Together, these results suggest that down-regulation of SPI-1 genes in rpsD* and rpsL* strains is mainly due to reduced levels of HilD protein.

### Non-optimal translational fidelity promotes degradation of HilD by Lon

**Figure 6.**
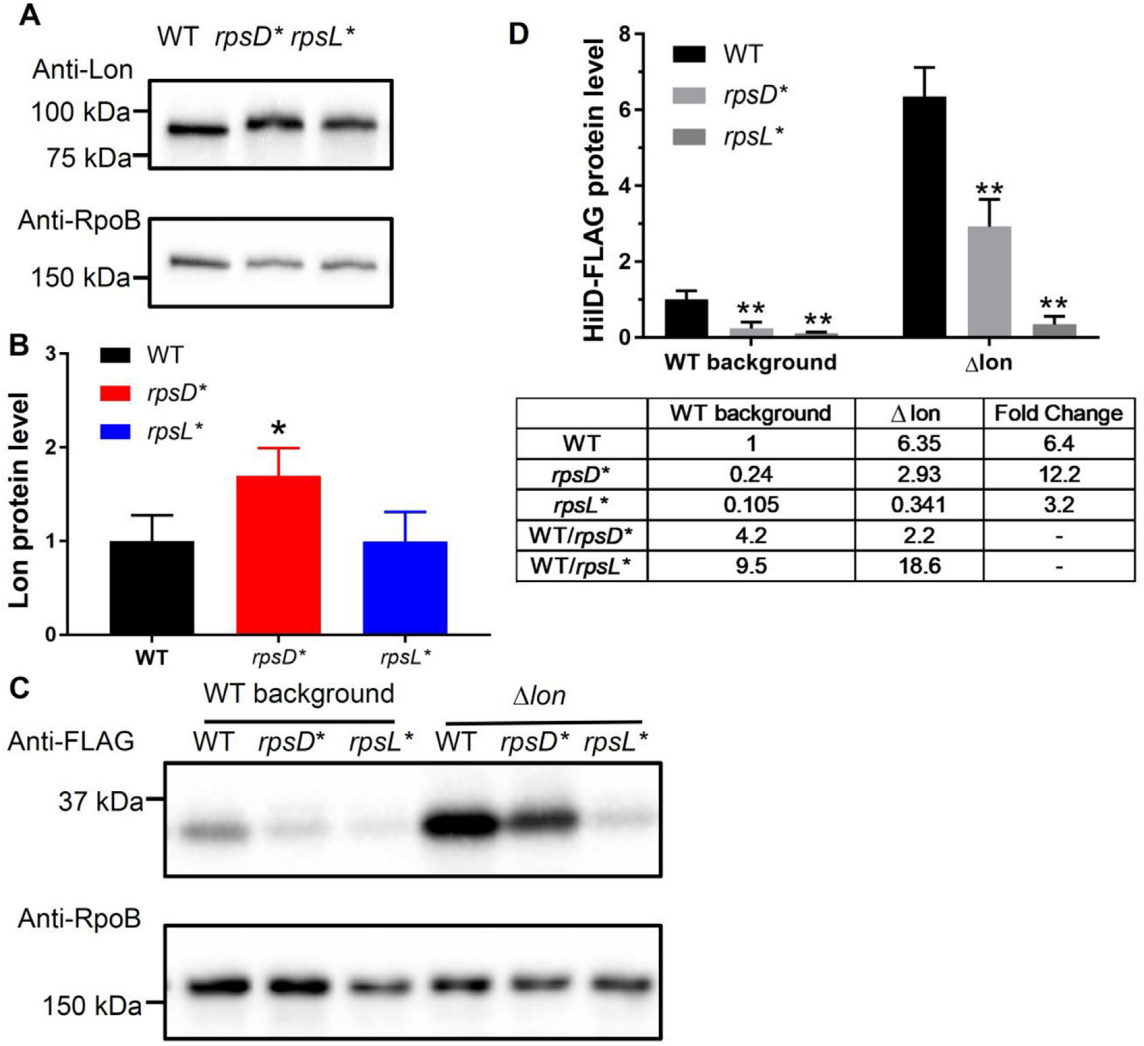
Non-optimal translational fidelity destabilizes HilD by enhancing Lon activity. (**A, B**) Western blotting against Lon from early-stationary phase cells grown in LB at 37°C. The protein level of Lon is increased in the rpsD* strain, but unaffected in the rpsL* strain compared with the WT. (**C, D**) Deleting *lon* increased the HilD protein level in the rpsD* and rpsL* strains. *n* = 3. *P < 0.05; **P < 0.01 determined using one-way ANOVA. Error bars represent one standard deviation.

A previous study has shown that HilD is degraded by the Lon protease in *Salmonella* (63). We confirmed that deleting *lon* abolished degradation of HilD in the WT, rpsD* and rpsL* S. Typhimurium strains (Figure 5E), suggesting that Lon is the primary protease responsible for destabilization of HilD. We next determined the Lon protein level using Western blotting, and found that Lon was increased in the rpsD* mutant compared with the WT, but remained unchanged in rpsL* (Figure 6B and C). Deleting *lon* increased the HilD protein level in all three strains, with the highest fold-increase observed in the rpsD* strain (Figure 6D and E). This suggests that enhanced degradation by Lon is partially responsible for the reduced HilD protein level in rpsD*.

### Down-regulation of *fliZ* reduces SPI-1 expression in rpsD*

Previous work has shown that a flagellar gene *fliZ* activates HilD post-translationally to enhance *hilA* expression (64). Our RNA-Seq results revealed that flagellar genes, including *fliZ*, were among the most significantly down-regulated in the rpsD* and rpsL* strains (Supplementary Tables S2–S5). Consistent with down-regulation of flagellar genes, we verified that swimming motility was reduced in both the rpsD* and rpsL* strains compared with the WT (Supplementary Figure S7).

**Figure 7.**
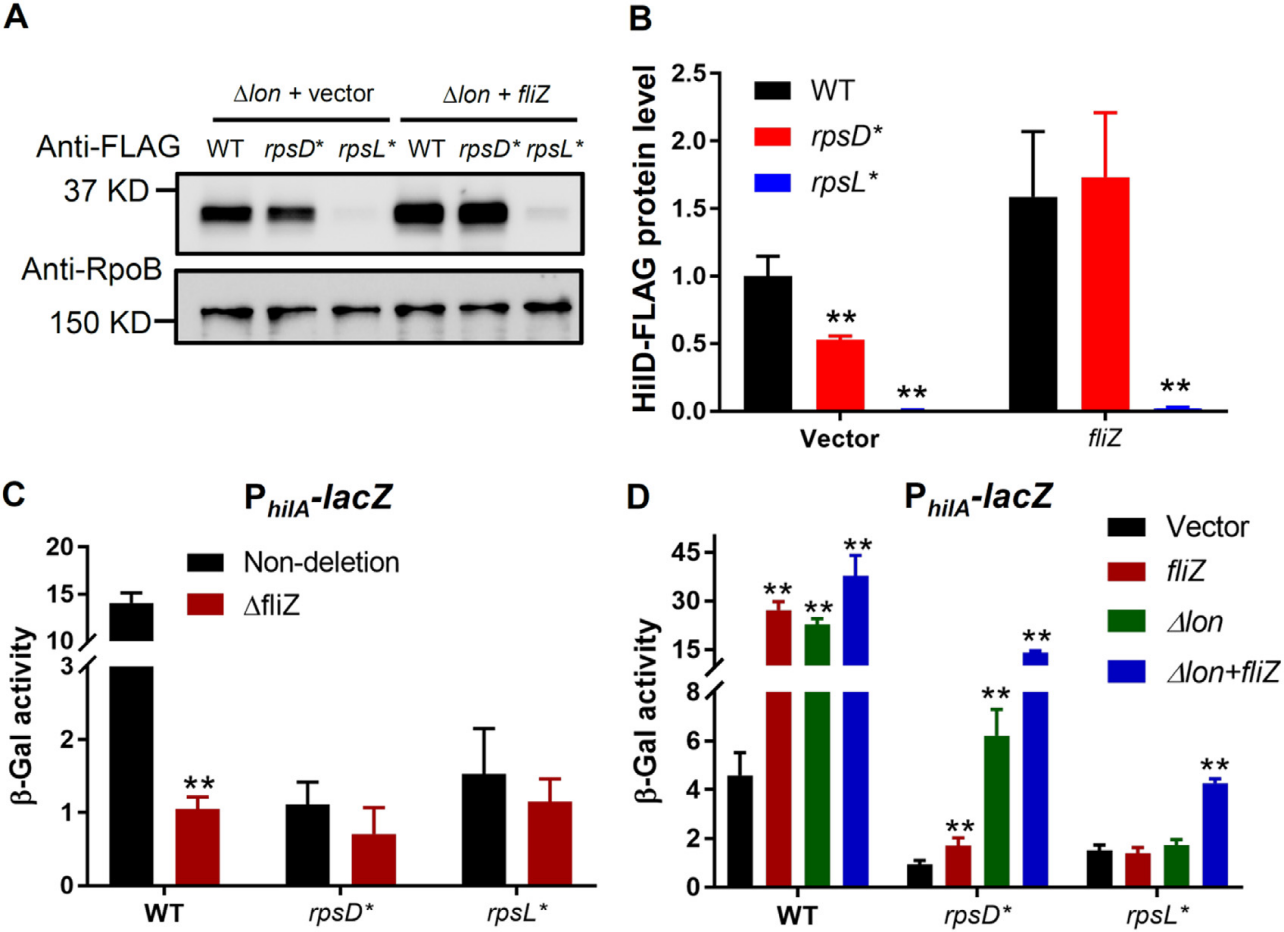
Overexpressing FliZ improves HilD activity in the absence of Lon. (**A**) *Salmonella* strains lacking *lon* were grown to early-stationary phase in LB media at 37°C with or without FliZ overexpression (induced by 10 μM IPTG), and Western blotting against HilD-FLAG and RpoB was performed. (**B**) Quantitation of results in (A). *n* = 3. (**C, D**) *S*. Typhimurium strains carrying a *lacZ* reporter for *hilA* promoter activity were grown to early-stationary phase in LB media at 37°C, and the β-galactosidase activity was determined. Deleting *fliZ* decreases *hilA* promoter activity in the WT. Overexpressing *fliZ* and deleting *lon* display additive effects to enhance *hilA* activity. *n* = 3. **P < 0.01 determined using one-way ANOVA. Error bars represent one standard deviation.

Our previously experiments revealed that deleting Lon partially restored the protein level of HilD in the rpsD* strain (Figure 6D). Further overexpression of *fliZ* in the Δ*lon* background fully restored HilD protein level in rpsD* to the WT (Figure 7A and B), presumably due to the positive autoregulation of HilD expression. We next tested the effect of *fliZ* on *hilA* expression. Deleting *fliZ* in the WT reduced the promoter activity of *hilA* 10-fold (Figure 7C), supporting the positive regulation of SPI-1 genes by FliZ. In the rpsD* strain, overexpressing FliZ increased the promoter activity of *hilA* 2-fold, but deleting *lon* further increased *hilA* expression 8-fold (Figure 7D). These data thus suggest that down-regulation of *fliZ*, combined with increased Lon activity, are mainly responsible for the reduced expression of SPI-1 genes in the error-prone rpsD* mutant. In rpsL*, overexpressing FliZ or deleting *lon* alone did not improve *hilA* expression. When combining FliZ overexpression and *lon* deletion in rpsL*, hilA expression was increased 3-fold, although still at a much lower level than the WT. It appears that Lon and FliZ only modestly contribute to the down-regulation of SPI-1 genes in rpsL*.

## DISCUSSION

The role of translational fidelity in bacterial pathogenesis is poorly understood. Our work here revealed that either increasing or decreasing translational fidelity down-regulates expression of virulence genes in *Salmonella* and impairs its interactions with the host. We used zebrafish as a vertebrate host because it is a well-established model of *S*. Typhimurium infection and its transparency allows real-time visualization of the infection process in live animals. Unlike the *Salmonella* mouse models, zebrafish develop intestinal infection against the backdrop of endogenous microbiota. *Salmonella* infection in zebrafish has been studied both in larval and adult fish, and in the context of innate and adaptive immunity (65–67) as well as pathogen-specific virulence factors (68,69).

It is intriguing that an increase or decrease in translational fidelity each leads to similar effects on expression of SPI-1 genes. Both increasing and decreasing ribosomal errors enhance degradation of a key SPI-1 regulator HilD by Lon protease (Figures 5 and 6). Increased translational errors in rpsD* may cause protein misfolding and activate heat-shock proteins, such as Lon (Figure 6B and Supplementary Figure S8). On the other hand, although decreasing translational errors in rpsL* does not affect the Lon protein level (Figure 6B), it may enhance Lon activity by reducing the amount of misfolded protein substrates for the protease (Supplementary Figure S8). In addition to regulation at the level of protein degradation, altering translational fidelity may also affect the expression and activity of HilD. We show here that a reduced level of *fliZ* in the error-prone rpsD* strain mitigates HilD activity and expression of SPI-1 genes (Figure 7). Our previous work showed that increased translational errors by the rpsD* mutation reduces expression of flagellar genes by up-regulating a small RNA DsrA in *E. coli* (70). An elevated level of translational errors may thus down-regulate *fliZ* via a similar mechanism in *Salmonella*. Our results here indicate that an increased activity of Lon and down-regulation of *fliZ* are major contributors for the reduced level of HilD and SPI-1 expression in rpsD*, although other pathways of transcriptional regulation are not completely ruled out (Supplementary Figure S8). In contrast, overexpression of FliZ and deletion of Lon only modestly restores *hilA* expression, suggesting that other unknown regulatory pathways are largely responsible for the SPI-1 defect in rpsL*. It is possible that an unknown transcriptional regulator of *hilD* is defective in rpsL*. Alternatively, specific codons or secondary structures of the *hilD* mRNA could prevent efficient translation of HilD by the rpsL* mutant ribosome.

Maintaining an optimal level of translational fidelity is a delicate process and needs to be carefully balanced. Increasing evidence shows that such balance can be frequently perturbed by environmental changes (5,30,71). Recent studies also show that translational errors are heterogeneous among individual cells within a genetically-identical population (42,72). Our work here has revealed that a fine balance of translational errors is critical for the virulence and host interactions of a bacterial pathogen. In future work, it will be intriguing to investigate how host conditions may affect the level and heterogeneity of translational errors in bacterial pathogens *in vivo*, and how variation of translational errors affect host interactions and survival of individual bacterial cells.

## DATA AVAILABILITY

GEO accession for RNA sequencing data: GSE123195.

## Supplementary Material

gkz229_Supplemental_Files
