# Supplementary material for: Optimal translational fidelity is critical for Salmonella virulence and host interactions
Source: Nucleic Acids Res. 2019 Apr 3;47(10):5356–67. doi: 10.1093/nar/gkz229 (PMC6547416; doi:10.1093/nar/gkz229)
Supplement: gkz229_Supplemental_Files [file gkz229_supplemental_files.zip › Supplemental material_Fan et al.pdf]

Optimal translational fidelity is critical for *Salmonella* virulence and host interactions

Yongqiang Fan[1,2,4]*, Laurel Thompson[2], Zhihui Lyu[3], Todd A. Cameron[2], Nicholas R. De Lay[2], Anne Marie Krachler[2]*, Jiqiang Ling[2,3]*

[1] College of Life and Health Sciences, Northeastern University, Shenyang 110819, People's Republic of China
[2] Department of Microbiology and Molecular Genetics, McGovern Medical School, University of Texas Health Science Center, Houston, TX 77030, USA
[3] Department of Cell Biology and Molecular Genetics, The University of Maryland, College Park, MD 20742, USA
[4] Corrosion and Protection Division, Shenyang National Laboratory for Materials Science, Northeastern University, Shenyang 110819, People's Republic of China

*Correspondence should be addressed to:

Yongqiang Fan: +86 (024) 8365-6108; Email: fanyongqiang@mail.neu.edu.cn
Anne Marie Krachler: +1 (713) 500-5465; Email: Anne.Marie.Krachler@uth.tmc.edu
Jiqiang Ling: +1 (301) 405-1035; Email: jling12@umd.edu

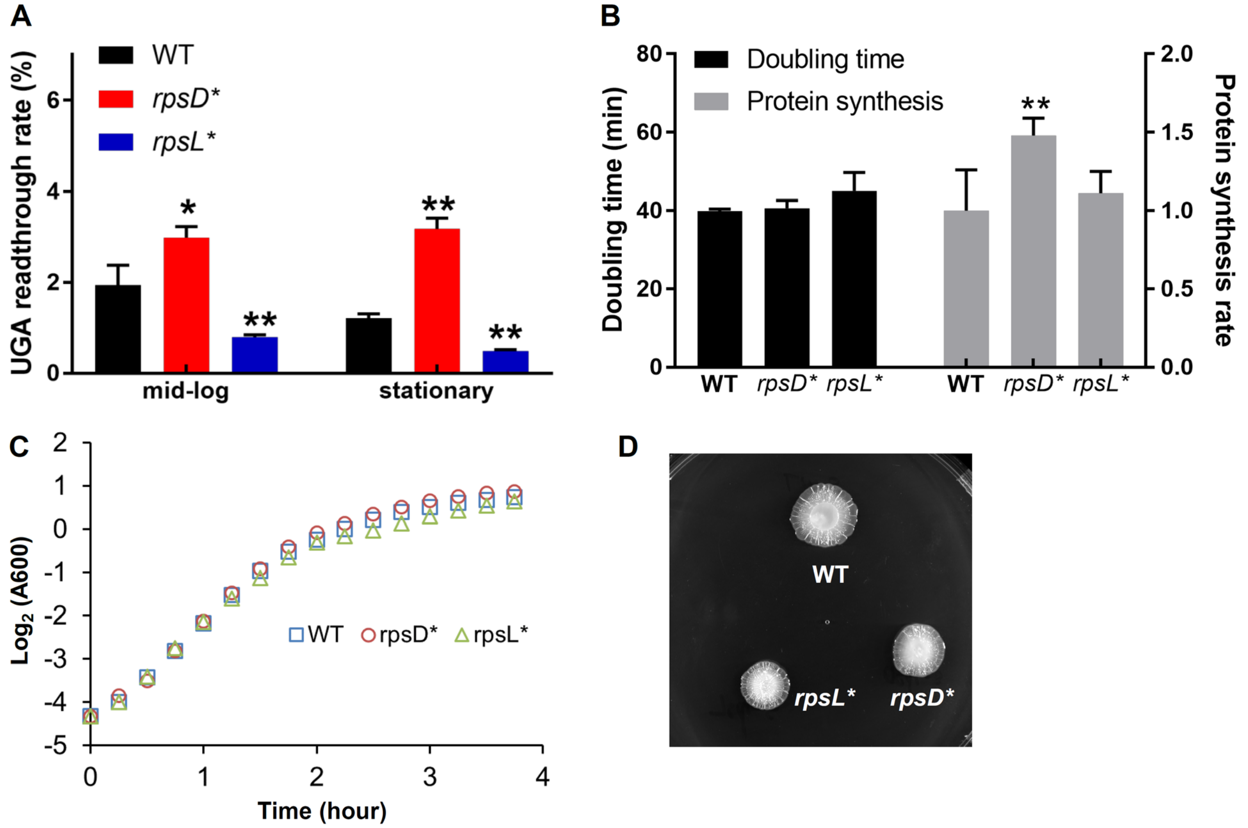

Figure S1. Error, growth, and protein synthesis rates of *Salmonella* variants. (**A**) A dual-fluorescence reporter was used to determine the UGA readthrough rates of *S.* Typhimurium cells at mid-log and stationary (OD_{600} ~2.5) phases. (**B, C**) The *rpsD** and *rpsL** mutants display little growth defect in LB media at 37 °C. The protein synthesis rates were measured with production of a reporter yellow fluorescence protein. (**D**) Colony morphology of *Salmonella* cells grown on LB agar at 37 °C after 96 hours. The smaller colony sizes of *rpsD** and *rpsL** may result from motility defects. Error bars represent one standard deviation.

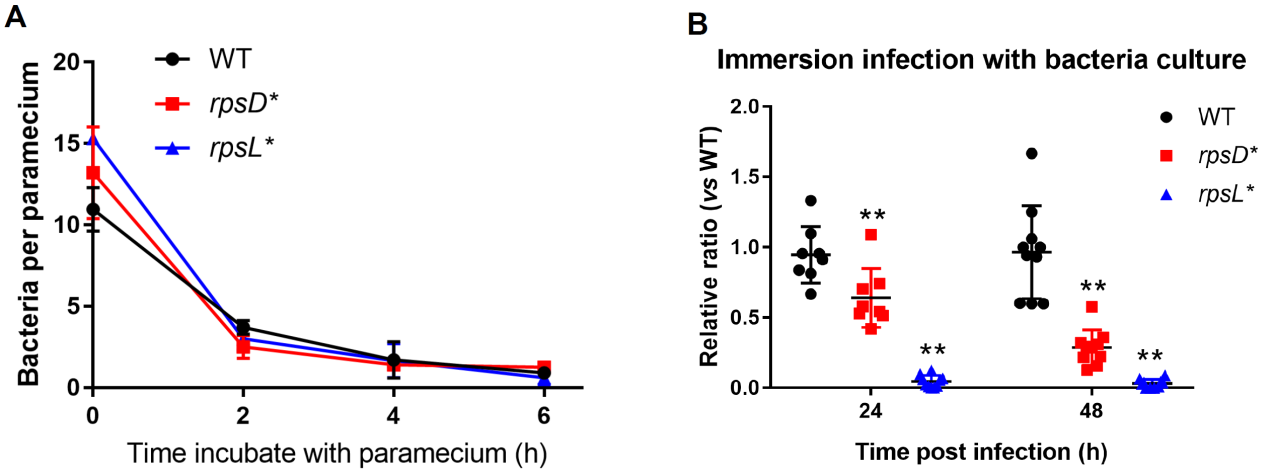

Figure S2. Infection of zebrafish with S. Typhimurium variants. (**A**) Survival rates of *S.* Typhimurium variants within paramecia were determined by CFU. (**B**) Zebrafish were infected with free *S.* Typhimurium cells in competition experiments, and the number of WT, *rpsD**, and r*psL** cells recovered from fish following infection were determined by CFU. ** $P < 0.01$ determined using one-way ANOVA. Error bars represent one standard deviation.

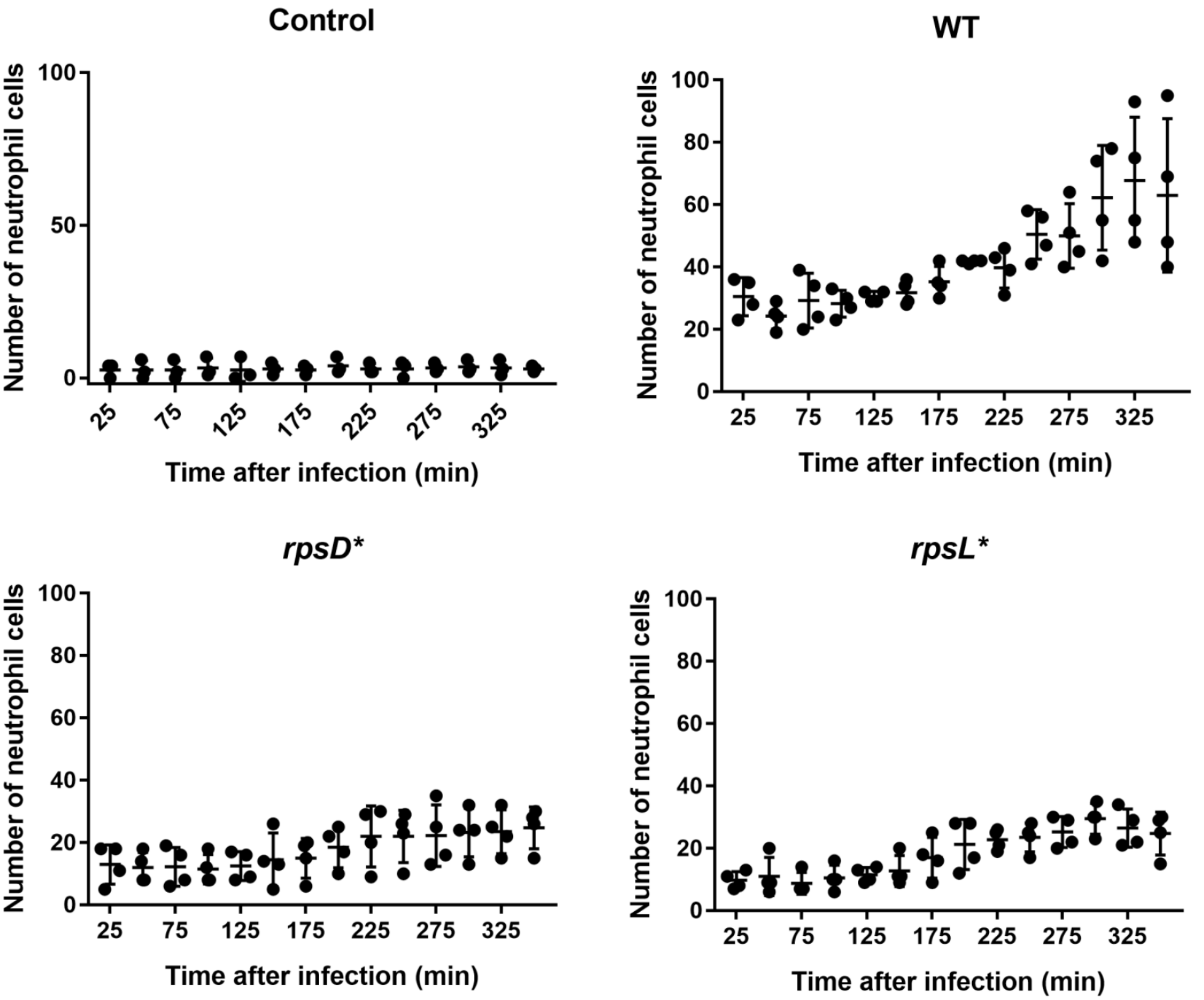

Figure S3. Recruitment of neutrophils into zebrafish intestine by S. Typhimurium. WT, *rpsD**, and *rpsL** cells carrying mCherry (red fluorescence) grown to early-stationary phase in LB at 37 °C were delivered to zebrafish via paramecia. The number of neutrophils (green fluorescence) recruited to the gut were determined over time. Error bars represent one standard deviation. Control: uninfected fish.

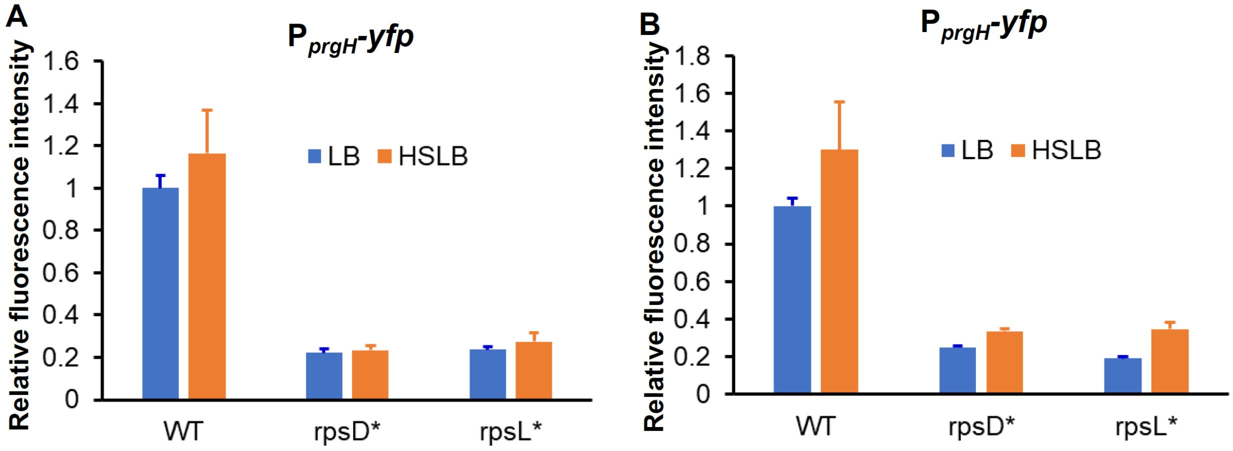

Figure S4. Expression of *prgH* at different salt concentrations. A *yfp* reporter is under the control of the *prgH* promoter as in Figure 4D. *Salmonella* strains were grown at 37 °C to mid-log (**A**) or early-stationary (**B**) phase in LB (5 g/l NaCl) or HSLB (10 g/l NaCl). The *prgH* promoter activity is similar in LB and HSLB. In both media, the *rpsD** and *rpsL** strains exhibit significantly reduced levels of *prgH* promoter activity compared with the WT. n = 3. Error bars represent one standard deviation.

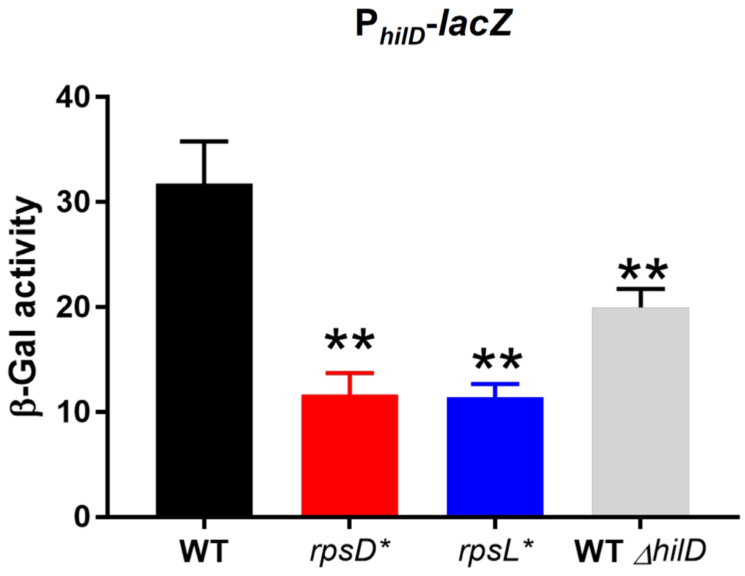

Figure S5. HilD is autoregulated at the transcriptional level. A *lacZ* reporter is under the control of the *hilD* promoter as in Figure 4D. *Salmonella* strains were grown in LB at 37 °C to early-stationary phase, and the β-galactosidase activity was determined. n = 3. ** $P < 0.01$ determined using one-way ANOVA. Error bars represent one standard deviation.

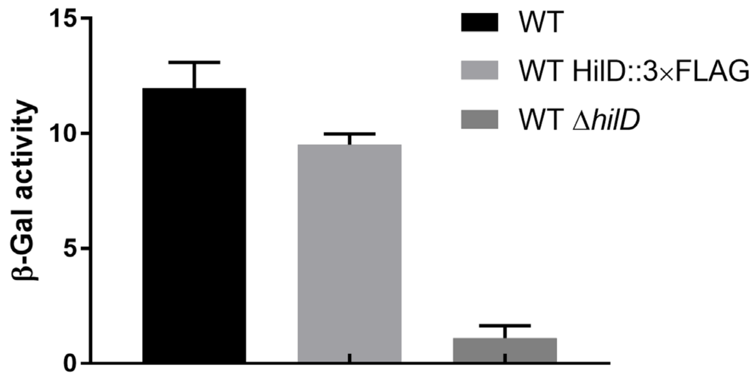

Figure S6. FLAG-tagged HilD is functional *in vivo*. A *lacZ* reporter is under the control of the *hilD* promoter as in Figure 4D. *Salmonella* strains were grown in LB at 37 °C to early-stationary phase, and the β-galactosidase activity was determined. The *hilD* deletion strain shows no detectable *hilA* promoter activity, whereas the HilD::3xFLAG strain shows 80% of the WT *hilA* promoter activity. n = 3. Error bars represent one standard deviation.

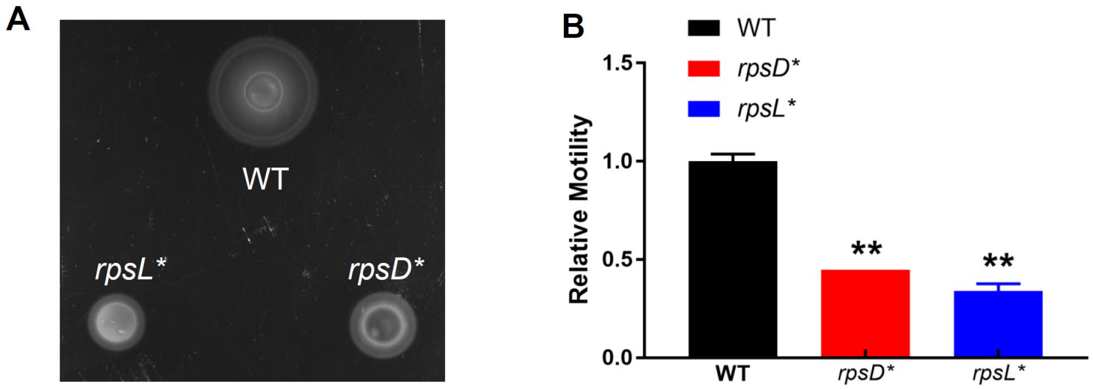

Figure S7. Non-optimal translational fidelity reduces motility in *Salmonella*. (**A**) A swimming motility assay reveals that *rpsD** and *rpsL** strains are defective in motility. (**B**) Quantitation of **A**. n = 3. ** P < 0.01 determined using one-way ANOVA. Error bars represent one standard deviation.

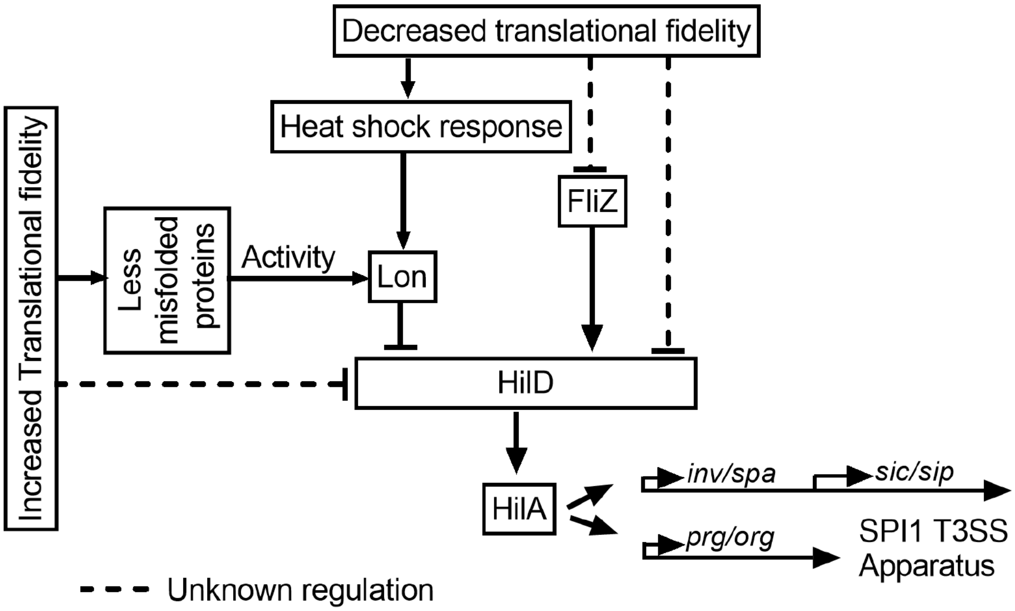

Figure S8. Model for the role of translational fidelity in SPI-1 expression. An optimal translational fidelity is key for expression of SPI-1 genes and maintenance of *Salmonella* virulence. A decreased level of translational fidelity (e.g., in *rpsD**) activates expression of the heat shock protease Lon, which degrades HilD and represses expression of SPI-1 genes. Decreased translational fidelity also downregulates expression of *fliZ* gene, which is required for activation of HilD. On the other hand, increased translational fidelity results in less misfolded protein and frees Lon to degrade HilD. Other unknown regulatory pathways also contribute to reduced SPI-1 expression when translational fidelity is increased. Therefore, both decreased and increased translational fidelity converged to down-regulate SPI-1 genes.

Table S1. Plasmids and strains

Plasmid	Source
pZS-P$_{tet}$-m-y	(1)
pZS-P$_{tet}$-m-TGA-y	(1)
pZS-P$_{hilA}$-lacZ	This study
AG1(pCA24N, -gfp)	(2)
AG1-*hilD*	This study
pZS-P$_{hilD}$-lacZ	This study
ASKA-fur	(2)
AG1-*fliZ*	This study
pKD46	(3)
pKD3	(3)
pZS-P$_{tet}$-mCherry-Chl	This study
pZS-P$_{tet}$-yfp-Spc	This study
PCDF	Lab stock
pZS*-11	(4)
pZS-thyA	This study
Strains	Source
WT	ATCC® 14028™
*rpsD**	This study
*rpsL**	This study
WT *ΔhilD*	This study
rpsD ΔhilD*	This study
rpsL ΔhilD*	This study
WT *Δlon*	This study
rpsD Δlon*	This study
rpsL Δlon*	This study
WT *ΔfliZ*	This study
rpsD ΔfliZ*	This study
rpsL ΔfliZ*	This study
WT *ΔhilE*	This study
rpsD ΔhilE*	This study
rpsL ΔhilE*	This study
WT insert::P*tet*-mCherry-Chl	This study
*rpsD** insert::P*tet*-mCherry-Chl	This study
*rpsL** insert::P*tet*-mCherry-Chl	This study
WT insert::P*tet*-yfp-Spc	This study
*rpsD** insert::P*tet*-yfp-Spc	This study
*rpsL** insert::P*tet*-yfp-Spc	This study
WT *HilD::3 ×FLAG*	This study
rpsD HilD::3◊FLAG*	This study
rpsL HilD::3◊FLAG*	This study

References

1. Fan, Y., Evans, C.R., Barber, K.W., Banerjee, K., Weiss, K.J., Margolin, W., Igoshin, O.A., Rinehart, J. and Ling, J. (2017) Heterogeneity of Stop Codon Readthrough in Single Bacterial Cells and Implications for Population Fitness. *Mol Cell*, **67**, 826-836 e825.

2. Kitagawa, M., Ara, T., Arifuzzaman, M., Ioka-Nakamichi, T., Inamoto, E., Toyonaga, H. and Mori, H. (2005) Complete set of ORF clones of Escherichia coli ASKA library (a complete set of E. coli K-12 ORF archive): unique resources for biological research. *DNA research : an international journal for rapid publication of reports on genes and genomes*, **12**, 291-299.

3. Datsenko, K.A. and Wanner, B.L. (2000) One-step inactivation of chromosomal genes in Escherichia coli K-12 using PCR products. *Proceedings of the National Academy of Sciences of the United States of America*, **97**, 6640-6645.

4. Subramaniam, A.R., Pan, T. and Cluzel, P. (2013) Environmental perturbations lift the degeneracy of the genetic code to regulate protein levels in bacteria. *Proceedings of the National Academy of Sciences of the United States of America*, **110**, 2419-2424.
